# Human Babesiosis Caused by *Babesia venatorum*, Russia, 2024

**DOI:** 10.3201/eid3109.250319

**Published:** 2025-09

**Authors:** Olga P. Zelya, Irina V. Kukina, Ludmila S. Karan, Elena A. Krasilovskaya, Vadim V. Garin

**Affiliations:** First Moscow State Medical University (Sechenov University), Moscow, Russia (O.P. Zelya, I.V. Kukina, V.V. Garin); Research Center of Neurology, Moscow (L.S. Karan); Centre for Strategic Planning of the Federal Medical and Biological Agency, Moscow (L.S. Karan); Sayanogorsk Interdistrict Hospital, Sayanogorsk, Russia (E.A. Krasilovskaya)

**Keywords:** *Babesia venatorum*, parasites, vector-borne infections, human babesiosis, tickborne disease, Siberia, Russia

## Abstract

We report a case of acute babesiosis in a splenectomized 63-year-old man in Siberia, Russia. We confirmed the causative agent, *Babesia venatorum*, by PCR. Our study demonstrated a change in the structure of the parasite population, from single parasite invasion of erythrocytes to multioccupancy, without an increase in parasitemia level.

Babesiosis is an emerging tickborne infection caused by intraerythrocytic protozoa. To date, researchers have described more than 50 cases of babesiosis in humans in Europe, almost always fulminant in splenectomized patients and typically attributed to *Babesia divergens*. Some recent reports also describe several cases of human infection with *B. venatorum*, associated with milder infections than those caused by *B. divergens* (*1*). Researchers have also described sporadic cases of babesiosis caused by infection with *Babesia microti* and *B. divergens* in Asia-Pacific regions (*2*,*3*), but the practically asymptomatic course of the human infection with *B. venatorum* is more common (*4*). Although reports have noted detection of *Babesia* spp. DNA in *Ixodes persulcatus* ticks in Siberia (*5*), cases of human infections have yet to be reported in that region of Russia.

We report the case of a 63-year-old man who resided in a forested, mountainous area of Khakassia, East Siberia, Russia, and had undergone splenectomy. On September 30, 2024, the man sought treatment for an influenza-like syndrome with signs and symptoms that included a fever of 38°C, severe general weakness, darkening of urine, a decrease in diuresis, jaundice, dyspnea, and stomachache. Attending physicians admitted the patient to the hospital on October 2, 2024 (Table). The patient reported no awareness of a tick bite and had received no blood products in the previous 3 months.

**Table:** Characteristics of case-patient in study of human babesiosis caused by *Babesia venatorum*, Russia, 2024*

Tests performed	Results by date						
Oct 3	Oct 7	Oct 10	Oct 14	Oct 22	Nov 8	Reference range
Hematologic parameters							
Hemoglobin, g/dL	11.2	9.3	11.6	11.6	12.0	15.5	11.7–18.0
Hematocrit, %	32.0	27.0	34.8	32.7	36.1	46.8	35–52
Erythrocytes, ×10^12^ cells/L	3.48	3.01	3.96	3.51	3.85	5.1	3.8–6.1
RBCN, L	10:100	4:100	7:100	NA	NA	NA	0:100
Platelets, ×10^9^/L	21	127	154	346	230	217	150–450
Leukocytes, ×10^9^ cells/L	4.8	8.3	7.8	5.7	9.1	10.4	4–11
Parasitemia level, %†	2.43	2.91	1.56	0	0	0	0
Biochemistry							
Alanine aminotransferase, U/L	7.4	22.0	NA	21.0	16.0	NA	≤40
Aspartate aminotransferase, U/L	27.0	67.0	NA	25.0	30.0	NA	≤40
Total bilirubin, μmol/L	84.9	38.1	NA	11.8	9.0	NA	3.1–16.9
C-reactive protein, mg/L	1.3	0.7	NA	1.6	4.0	NA	0–0.5
Urea, μmol/L	na	7.5	NA	3.3	6.0	NA	2.5–8.3
Creatinine, mcM/L	97.0	91.0	NA	111.0	107.0	NA	53–106
Glucose, μmol/L	25.6	14.6	NA	8.4	7.5	NA	3.3–5.5
Total protein, g/L	56.0	55.0	NA	68.5	93.0	NA	62–81

*NA, not available; RBCN, red blood cell normoblasts. †Percentage of erythrocytes that were infected.

Blood smears obtained on hospital admission tested positive for *Plasmodium* spp. However, because the patient reported no recent travel to malaria-endemic areas, we sent the blood samples for retesting at Sechenov University (Moscow, Russia), where results confirmed babesiosis. We then examined the blood smears after Romanovsky-Giemsa staining, noting the paired forms diverging at a wide angle (≤180°), which is characteristic of both *B. divergens* and *B. venatorum* (Appendix Figure 1).

We screened DNA samples extracted from the blood smears for *B. divergens*, *B. microti*, and *B. venatorum* by PCR, using methods described previously (*6*). We partially sequenced the 18S rRNA gene (1,112 bp; GenBank accession no. PV086113) (*5*). We aligned, compared, and analyzed the resulting nucleotide sequences and reference sequences downloaded from GenBank by using MEGA X (*7*). We also reconstructed a phylogenetic tree (Appendix Figure 2). Using forward and reverse primers form 18S RNA of *Babesia* spp. from Europe, we were able to detect only *B. venatorum* DNA.

We started etiotropic treatment for the patient 3 days from the time we initially detected intraerythrocytic parasites and identified the causative agent as *Babesia* spp. The patient responded to therapy (clindamycin and quinine sulfate), and by day 9 of treatment, parasites were no longer detectable with microscopy (Table). We noted that the ratio of erythrocytes invaded by >1 trophozoite changed as the infection progressed. Four days after symptom onset, single trophozoites and pairs (figure 8 pattern) predominated. On the 8th day of infection, with the same level of parasitemia, almost half of the infected erythrocytes contained ≥4 trophozoites (Figure). Parasites continued to divide intensively but did not leave the host cell. Prior research has noted multiple parasites present in individual erythrocytes during fulminant infections in humans (*8*) and in heavily infected in vitro cultures (*9*). Results of such studies suggests that multioccupancy of trophozoites in the erythrocyte prevents a sharp increase in parasitemia and helps to preserve the parasite population in the host (*9*). However, the phenomenon we observed occurred at both high (>23%) and low (<3%) parasitemia.

**Figure:**
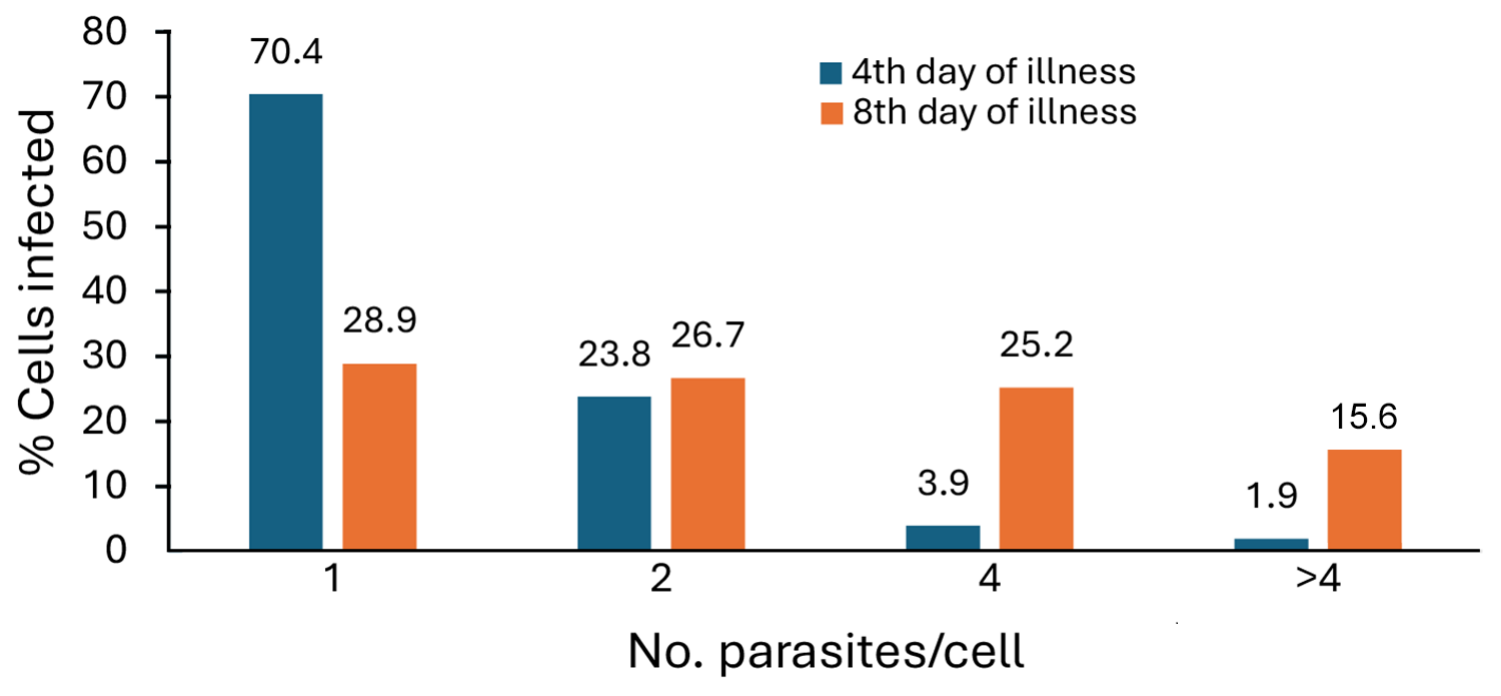
Dynamics of the parasite population in erythrocytes in case-patient in study of human babesiosis caused by *Babesia venatorum*, Russia, 2024. As the infection progressed with constant parasitemia (<3%), 4 main populations of infected red blood cells were seen: 1 parasite, 2 parasites, 4 parasites, and >4 multioccupancy (>4 parasites).

Our study of this unique case of human babesiosis in Siberia, Russia, provides molecular evidence that the etiologic agent was *B. venatorum.* Prior research noted the Asia variant of *B. venatorum* in asymptomatic persons from China (*4*). *B. divergens* infection in splenectomized humans could lead to death, even with timely treatment (*1*). However, the similar course and positive outcome of babesiosis in our patient resembled those in cases previously reported in Europe (*1*), suggesting that the causative agent of the disease was *B. venatorum*, which we confirmed by PCR. 

Our patient resided in a village located in a forested area, which is a natural habitat for ticks. The man’s work involved staying in the forest, again increasing his risk for tick bites. The fact that the patient did not notice a tick bite is not unusual. Only 50% to 70% of patients with tickborne diseases recall being bitten by a tick (*2*). Considering the ability of *B. venatorum* to be transmitted transovarially and transstadially within *I. persulcatus* ticks, it is possible for a person to become infected through the bite of a tick nymph, which is smaller and less noticeable than the adult (*10*).

In conclusion, our case study revealed the potential risk of *B. venatorum* infection for persons living in Siberia, Russia. Clinicians should be aware that infection can occur as an influenza-like illness and may go unnoticed in immunocompetent persons. 

Appendix Additional information for human babesiosis caused by *Babesia* *venatorum*, Russia, 2024.
