# Supplementary material for: Human Babesiosis Caused by Babesia venatorum, Russia, 2024
Source: Emerg Infect Dis. 2025 Sep;31(9):1860–2. doi: 10.3201/eid3109.250319 (PMC12407196; doi:10.3201/eid3109.250319)
Supplement: Appendix — Additional information for human babesiosis caused by Babesia venatorum, Russia, 2024. [file 25-0319-Techapp-s1.pdf]

Article DOI: http://doi.org/10.3201/eid3109.250319

EID cannot ensure accessibility for supplementary materials supplied by authors. Readers who have difficulty accessing supplementary content should contact the authors for assistance.

Human Babesiosis Caused by *Babesia venatorum*, Russia, 2024

Appendix

Patient Demographics

Our case patient lived in Siberia (Russia), in a village located in a forested area; the patient's work involves staying in the forest; he did not notice a tick bite. The patient's initial clinical manifestations included: fever (38°C) and chills developed on September 30, 2024; hospitalized on October 02 because of fever, severe general weakness, dyspnea, stomachache, jaundice, and dark urine. His medical history reported diabetes and posttraumatic splenectomy. On admission to the hospital, a significant excess of normal values of total bilirubin and C-reactive protein and a sharp decrease in platelets were noted. Red blood cell counts were slightly reduced, but signs of dyserythropoiesis (normoblasts), anisocytosis and poikilocytosis, a shift in the leukocyte formula to the left with immature neutrophils (up to promyelocytes) were observed in the peripheral blood, but the total number was normal. As the treatment progressed, the blood counts returned to normal values

Etiotropic Therapy

The patient's therapy consisted of clindamycin (600 mg thrice daily, by intravenous infusion) and quinine sulfate (650 mg thrice daily, per os) for 15 d, from October 06 to 20, 2024. His fever subsided by day 3 of therapy; no parasites found by blood-smear examination after day 9 of therapy. On October 23, 2024, the patient was discharged in satisfactory condition after 3 negative blood tests.

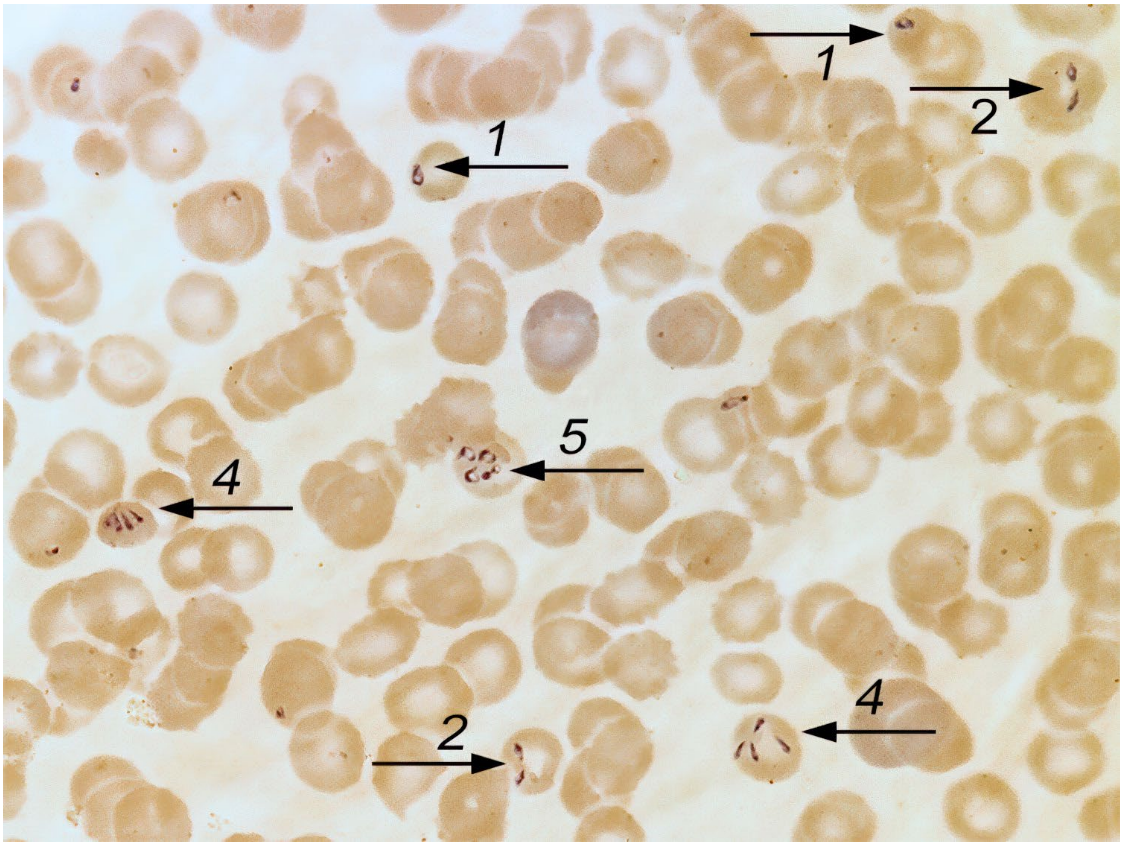

Appendix Figure 1. Romanovsky-Giemsa−stained thin blood film from a case of autochthonous human *Babesia venatorum* infection, confirmed by PCR, Siberia, Russia. Original magnification x1,250. There is clear polymorphism of parasite stages in a thin film due to the asynchronous nature of *Babesia* multiplication and egress from the erythrocytes. Arrows (1) indicate red blood cells containing single round forms of the parasite; (2) show typical paired pear-shaped forms; (4) show tetrad forms, and the arrow (5) shows multiple round-form parasites.

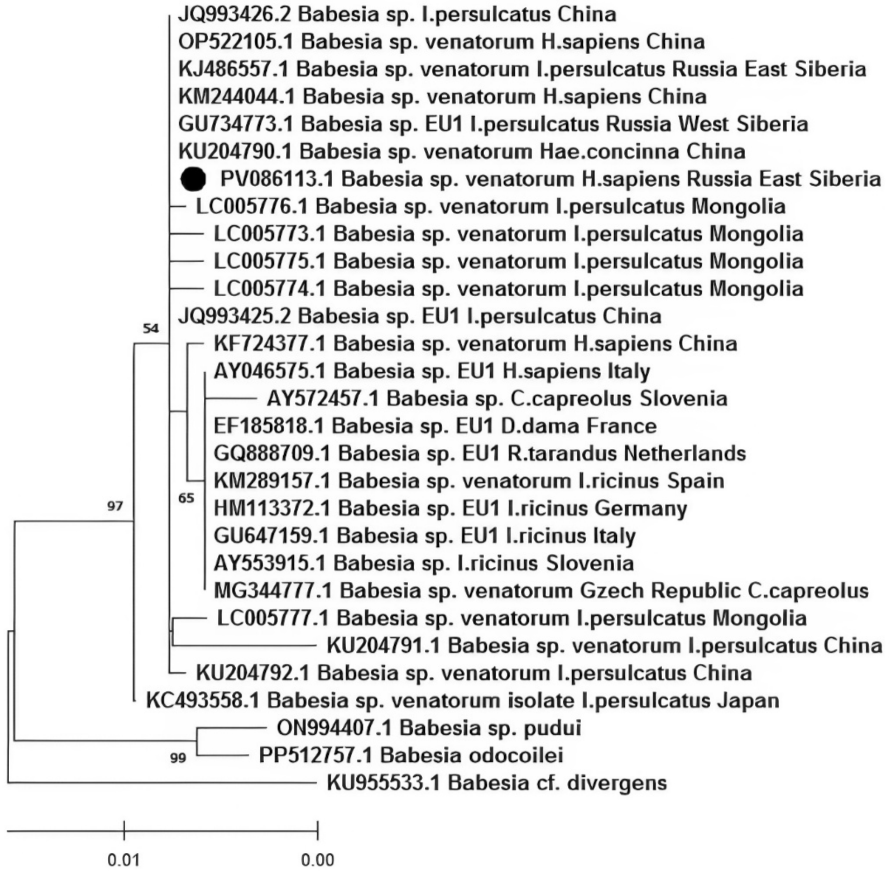

Appendix Figure 2. The phylogenetic tree constructed using the 1112 bp sequences of the *Babesia* spp. 18S rRNA gene. Phylogenetic tree was inferred using the maximum likelihood method and the T92+G model with 1,000 pseudoreplicates. Bootstrap replicates ≥54% are indicated. Sequences obtained in this study are denoted by black circle. The piroplasm 18S rRNA sequence from our patient has 99.9%–100% identity with *B. venatorum* previously detected in ticks in West and East Siberia, Russia (GenBank accession nos. GU734773, KJ486557) and in ticks and humans in some regions of People’s Republic of China (JQ993426, etc.). *B. venatorum* isolated in Europe form a separate cluster, although these samples differ by only 2 nucleotides in the analyzed region. Scale bar represents mean number of nucleotide substitutions per site.
